# DeepDRP: Dose-response predictions of drug pairs using deep learning based on data-driven feature representation and dose-response curve characteristics

**DOI:** 10.1371/journal.pone.0348908

**Published:** 2026-05-08

**Authors:** Mohammadamin Moragheb, Alireza Dehghan, Parvin Razzaghi, Sajjad Gharaghani

**Affiliations:** 1 Department of Bioinformatics, Kish International Campus, University of Tehran, Kish, Iran; 2 Department of Computer Engineering, Faculty of Technology and Engineering, Salman Farsi University of Kazerun, Kazerun, Iran; 3 Department of Computer Science and Information Technology, Institute for Advanced Studies in Basic Sciences (IASBS), Zanjan, Iran; 4 Laboratory of Bioinformatics and Drug Design (LBD), Institute of Biochemistry and Biophysics, University of Tehran, Tehran, Iran; Universita degli Studi di Siena, ITALY

## Abstract

Combination therapies have become a cornerstone of modern medicine, offering improved treatment outcomes and reduced side effects compared to monotherapies. However, the efficacy and safety of drug combinations depend heavily on the specific doses of each component, making the optimization of dosing regimens a crucial yet challenging task. Despite the importance of dose optimization, few computational methods address this challenge. Here, we propose DeepDRP, a novel approach that integrates two complementary models: the global model, which is trained using all available combinations, and the local model, which utilizes only samples with similar information to queries. The DeepDRP architecture comprises three main modules: the first module has three embedding networks to extract features from drugs, doses, and cell lines, and then predicts synergy using the fused knowledge. The second module constructs a graph using the input query and the set of similar retrieved training samples fed into a semi-supervised graph convolutional network to predict the synergy value. Finally, these two models are aggregated to have the final predicted value. To evaluate the proposed approach, it is applied to the NCI-ALMANAC dataset and O’Neil dataset. The obtained results denote that the proposed method achieves superior performance with respect to the other approaches.

## 1 Introduction

Drug dose-response prediction is a critical aspect of pharmacology and toxicology that focuses on understanding and predicting how an organism will respond to different doses or concentrations of a drug [1–4]. It involves the study of the relationship between the amount of a drug administered and the resulting biological response, which can range from therapeutic effects to adverse reactions [5–7].

The primary objective of drug dose-response prediction is to optimize the use of medications by determining the appropriate dose or concentration required to achieve the desired therapeutic outcome while minimizing the side effects. This process is essential in drug development, clinical pharmacology, and personalized medicine [8–12].

Drug dose-response prediction has broad applications in various fields. In drug development, it guides the selection of appropriate doses for pre-clinical and clinical trials, helping determine the maximum tolerated, minimum effective, and optimal therapeutic doses. In clinical pharmacology, it assists in designing personalized dosing regimens, taking into account individual patient factors such as age, weight, genetic variations, and organ function. Furthermore, drug dose-response prediction contributes to understanding drug mechanisms of action, drug interactions, and the development of combination therapies. It also plays a crucial role in toxicology, helping assess the safety profile of drugs and identifying potential toxic effects at higher doses or concentrations [13–23].

The drug dose-response curve is a fundamental tool to understand and predict how a drug affects an organism [24]. The dose-response curve, also known as a concentration-response curve, describes the relationship between the dose or concentration of a drug administered and the resulting biological response. The curve for a single drug typically has a characteristic sigmoidal (S-shaped) shape, and it provides valuable information about the potency and efficacy of a drug [25].

The dose-response curve helps determine a drug’s potency, which measures how much of the drug is needed to produce a specific effect [26]. The curve allows researchers to identify the drug’s effective dose or concentration range. The curve also helps evaluate the efficacy of a drug, which refers to the maximum response that can be achieved. It provides insights into how effectively the drug produces the desired response. The drug dose-response curve is a fundamental tool in pharmacology and toxicology that helps researchers and clinicians make informed decisions about drug dosing, efficacy, and safety [27].

Dose-response curves for drug combinations are often more complex than those for single drugs, potentially exhibiting multiple peaks or valleys, indicating complex interactions between the drugs. Recently, drug combinations have many real-world applications. Combination chemotherapy is a common approach in cancer treatment, and understanding the dose-response curves for drug combinations is crucial for optimizing treatment outcomes. Also, combination therapy is often used to treat infectious diseases, and analyzing dose-response curves for drug combinations can help identify the most effective treatment regimens [28]. Moreover, analyzing dose-response curves for drug combinations can help tailor treatment to individual patients based on their genetic profiles and medical histories [29]. Recent studies have explored hybrid deep learning architectures for prediction tasks in complex biological and societal systems. For example, a deep hybrid neural network was proposed for predicting COVID-19 hotspots by integrating heterogeneous data sources and capturing spatiotemporal dynamics [30].

Until now, many classic machine learning methods are applied to drug dose-response prediction, including random forest, support vector machine (SVM), gradient boosting, and neural network [7,13–23]. While ML methods have shown promising results in dose-response prediction, there are several challenges and limitations. High-quality, large-scale datasets are often limited, which can impact the performance of ML models. While some ML methods provide interpretable results, others can be difficult to interpret, making it challenging to understand the underlying mechanisms. ML models can suffer from overfitting, particularly when dealing with small datasets. ML methods may not incorporate domain-specific knowledge, which can lead to biased or inaccurate predictions. In most of the introduced methods, the prediction is done by utilizing dose-response curve characteristics. This paper introduces a new method that utilizes dose-response curve characteristics and learns a model using all available drug combinations.

The proposed method has three main steps: first, a deep learning-based model is learned using all available data. This model learns a feature extraction approach for the input-paired drugs and cell lines. In the second step, dose-response curve information is utilized to have another prediction. In this case, we have utilized the available tuples (i.e., paired drugs and the input cell line) in the training set, which are the same as the input with different doses, to predict the response for the input drugs’ doses. In this step, we have used the semi-supervised graph convolutional network (GCN). Finally, the two predicted outputs are fed into an MLP to aggregate the knowledge to have a final prediction. It is applied to the ALMANAC dataset [31] and O’Neil dataset to evaluate the strength of the proposed method. Also, three scenarios, like the comparable approaches, are designed to evaluate the strength of the proposed method. The results show that it performs better than comparable approaches in all scenarios.

The main contributions of this work are as follows

1- We propose DeepDRP, a novel framework that jointly models global drug–dose–cell line interactions and local dose-response curve information for drug combination synergy prediction.
2- We introduce a semi-supervised graph-based formulation of dose-response prediction, where known dose-response points are treated as labeled nodes and unseen dose combinations are inferred via graph convolution.
3- We develop an adaptive aggregation mechanism that seamlessly combines global and local predictions, allowing the model to operate effectively in both data-rich and data-scarce settings.
4- We demonstrate that the proposed approach achieves state-of-the-art performance using limited auxiliary information, outperforming or matching methods that rely on extensive multi-omics features.

This paper is organized as follows: the related works are reviewed first. Then, the proposed methods is given in section 3 in detail. In section 4, the experiments are given and analyzed, and finally, the obtained results are discussed, as well as the advantages and disadvantages of the proposed method.

## 2 Related work

The previous studies show that a single metric from a dose-response curve is insufficient for capturing the full scope of a drug’s sensitivity profile, which is crucial for determining the optimal dosage for patients. To address this limitation, researchers have explored using machine learning algorithms to predict the entire dose-response curve based on genetic data. Rahman et al. [32] introduced a novel approach, Functional Random Forest, developed to improve existing methods. This approach involves modifying the traditional Random Forest algorithm to directly predict the full dose-response curve rather than a single summary metric. The results have shown that Functional Random Forest outperforms existing methods in predicting dose-response curves, particularly when using functional data. This breakthrough has significant implications for personalized medicine, enabling the development of more effective treatment plans tailored to individual patients’ needs.

Another machine learning framework called comboFM [33] has been presented, which is designed to predict the responses of drug combinations in pre-clinical studies. The framework models drug interactions using a higher-order factorization machine [34] and leverages information from previous experiments to make accurate predictions, even with sparse data. The effectiveness of comboFM has been demonstrated through various prediction scenarios and experimental validation, including the discovery of a novel synergy between two drugs in lymphoma cells.

A powerful treatment modality, combination therapies, has been developed to overcome drug resistance and improve treatment efficacy. However, the experimental screening of all possible drug combinations has been made infeasible due to the rapidly increasing number of combinations. Machine-learning models have been proposed as a time- and cost-efficient solution to prioritize the most effective combinations for further validation. Nevertheless, the complexity of interaction patterns across multiple drug doses and cellular contexts has posed challenges to predicting combination effects [35].

A highly time-efficient method, comboLTR, has been introduced for learning complex, non-linear target functions that describe the responses of therapeutic agent combinations in various doses and cancer cell contexts [36]. The method has been based on polynomial regression via latent tensor reconstruction, utilizing a combination of recommender system-style features and chemical and multi-omics features as inputs. It has been demonstrated that comboLTR outperforms state-of-the-art methods in terms of predictive performance and running time, producing highly accurate results even in challenging scenarios where full dose-response matrices are predicted for new drug combinations with no available combination and monotherapy response measurements in any training cell line [36].

## 3 Proposed method

**Fig 1:**
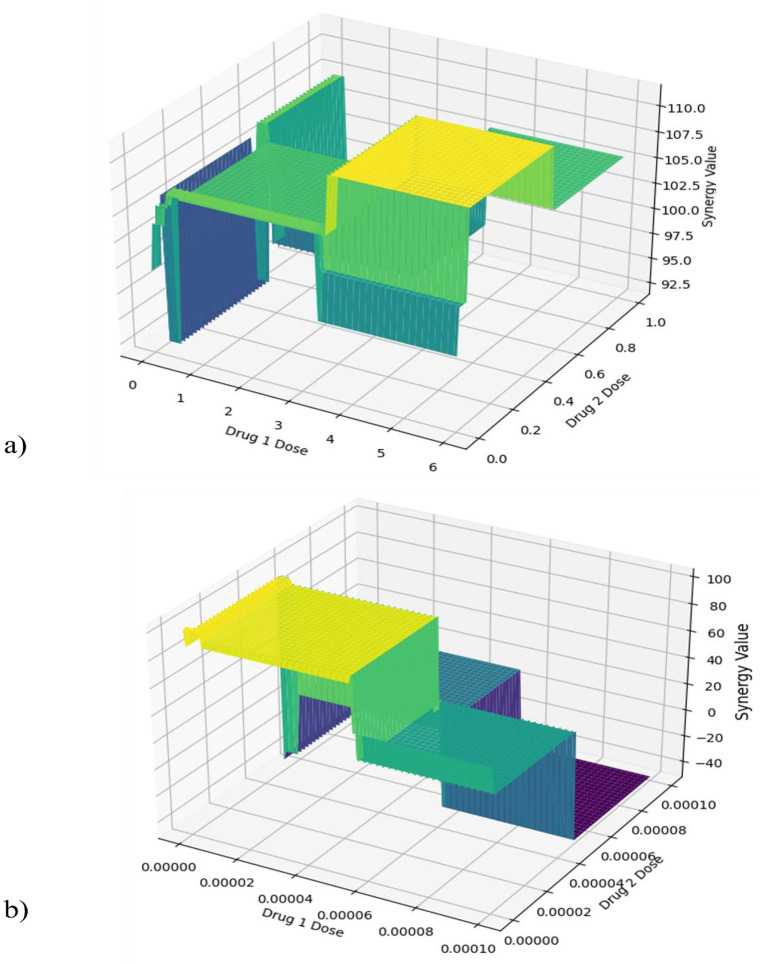
Synergy values of drug combinations. (a) Meshgrid representation of the synergy value between Chlorambucil and Exemestane at different doses. The x-axis represents the dose of Chlorambucil, the y-axis represents the dose of Exemestane, and the z-axis represents the synergy value. ‘2-Fluoro Ara-A’, ‘Axitinib’. (b) Meshgrid representation of the synergy value between Chlorambucil and Exemestane at different doses. The x-axis represents the dose of Chlorambucil, the y-axis represents the dose of Exemestane, and the z-axis represents the synergy value.

In this section, the proposed method is explained in detail. To this end, first, the problem formulation is given. Then, the three building modules of the proposed method are given. Fig 1 shows the meshgrid representation of the synergy value between Chlorambucil and Exemestane and between Chlorambucil and Exemestane at different doses. It denotes that dose-response curves for drug combinations are different from the unidrug. Hence, we select to model these curves with the learnable non-linear function instead of using the known functional forms.

### 3.1 Problem formulation

Given $\overset{N}{\underset{i=1}{\left\{\left(\overset{\left(i\right)}{\underset{1}{d}}.\;\overset{\left(i\right)}{\underset{2}{d}}.\overset{\left(i\right)}{\underset{1}{r}}.\overset{\left(i\right)}{\underset{2}{r}}.c^{\left(i\right)}\right).s^{\left(i\right)}\right\}}}$ denotes the training dataset for dose-response prediction in drug combinations. In this case, $\left(\overset{\left(i\right)}{\underset{1}{d}}.\;\overset{\left(i\right)}{\underset{2}{d}}.\overset{\left(i\right)}{\underset{1}{r}}.\overset{\left(i\right)}{\underset{2}{r}}.c^{\left(i\right)}\right)$ indicates the input and $s^{\left(i\right)}$ shows the corresponding dose response for the input. In this problem, there are five inputs, including the first drug and its corresponding dose ($\overset{\left(i\right)}{\underset{1}{d}}.\overset{\left(i\right)}{\underset{1}{r}}$), the second drug and the corresponding dose ($\overset{\left(i\right)}{\underset{2}{d}}.\overset{\left(i\right)}{\underset{2}{r}}$), and the cell line ($c^{\left(i\right)}$). The main goal of the proposed model is to design a system that takes the input and predicts the paired drug synergy in the specified doses as an output.

As mentioned, the proposed model’s main contribution is utilizing the dose-response curve for each paired drug and the global knowledge of drug dose-response prediction by using all pairs. The proposed model has three main modules. The first module learns a prediction model using all available paired drugs and cell lines, called the global model. The second module fine-tuned the first module using the dose-response curve characteristic for the input data. The third module integrates these two modules to predict the final response value. In the following, each of these modules is explained in detail.

### 3.2 The first model: Global model

The first module includes three subnetworks: 1) a drug feature extraction network shared for both drugs, 2) a cell-line feature extraction network, and 3) a drug dose embedding network shared for both drugs. This module utilizes all data without considering the input paired drugs. In the following, each of these subnetworks are explained in detail.

#### 3.2.1 Drug feature extraction network.

**Fig 2:**
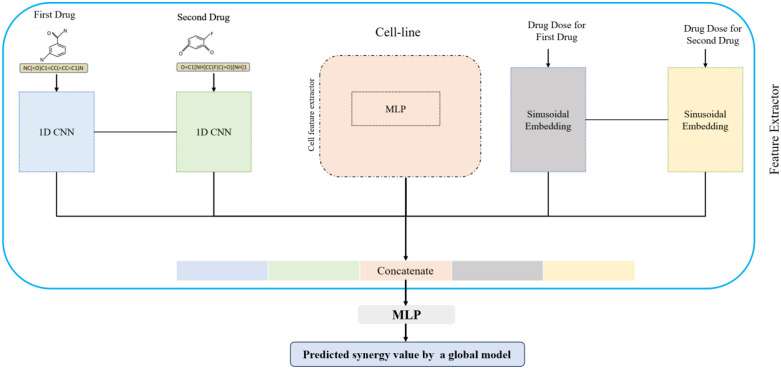
The overall schematic of the first module of the DeepDRP.

In this research, each drug is represented as a SMILES sequence, and to map it to an embedding space, a 1D-CNN is utilized, a powerful technique employed in computational drug discovery and cheminformatics (Fig 2). The core component of a 1D CNN is the convolutional layer. This layer applies a set of learnable filters or kernels to the input sequence. Each filter slides along the sequence and performs a convolution operation involving element-wise multiplication and summation, capturing local patterns and features. The filters are designed to have a small receptive field, allowing them to focus on nearby atoms or substructures in the molecular sequence. In the following, $E_{d}$ shows the drug embedding network.

#### 3.2.2 Drug dose embedding network.

Sinusoidal Embedding is a technique used in natural language processing (NLP) to encode sequential data, such as text or time series, into a continuous vector representation [37]. It can also be applied to drug dose sequences to effectively capture periodic patterns and trends.

Sinusoidal Embedding involves mapping the input data points to a high-dimensional space using sinusoidal functions. In the context of drug dose data, each data point represents a specific dose or concentration level. The sinusoidal functions introduce periodicity and capture patterns that may exist in the data. Sinusoidal Embedding maps the drug dose data to a higher-dimensional space, typically using a set of sine and cosine functions with different frequencies and phases. This transformation allows the model to capture complex patterns and relationships that may not be apparent in the original lower-dimensional space.

Each drug dose value is encoded using a sinusoidal function. If you have a sequence of drug doses [$d_{1}.d_{2}.\ldots .d_{n}$], the sinusoidal encoding for each dose $d_{i}$ can be calculated as follows:


$$E_{r}(d)=\left[\text{sin}\left(2\pi f_{1}d\right),\text{cos}\left(2\pi f_{1}d\right),\ldots ,\text{sin}\left(2\pi f_{c}d\right),\text{cos}\left(2\pi f_{c}d\right)]\right]$$ (1)

where $f_{1}$, $f_{2}$, …, $f_{c}$ are frequencies sampled from a logarithmic scale between the minimum and maximum frequencies. Using both sine and cosine functions, the model can capture both amplitude and phase information, allowing it to effectively represent and distinguish different dose levels.

#### 3.2.3 Cell-line feature extraction network.

In this paper, like ComboFM [33], the gene expression profiles of the cancer cell lines are used. These profiles are obtained from the rcellminer R package [38]. During the measurement of these profiles, the top 0.5% of genes with the highest variance across samples are selected, leading to 78 gene expression values per cell line. Then, these feature vectors are passed to a multi-layer perceptron to map these feature spaces into an embedded space. This network contains three fully connected layers which utilize ReLU as an activation function. In the following, $E_{c}$ shows the cell-line embedding network.

#### 3.2.4 Synergy prediction network.

After extracting the feature vector for the paired drugs, their corresponding doses, and the gene expression profile of the cell line, they are concatenated and fed into the MLP network to predict the synergy value of the paired drugs for the input cell line. The synergy prediction network is shown by $N_{s}$.

To train this network, the mean squared error is used as a loss function, which is defined as follows:


$${\left\|N_{s}\left(E_{d}\left(\overset{\left(i\right)}{\underset{1}{d}}\right).E_{d}\left(\overset{\left(i\right)}{\underset{2}{d}}\right).E_{r}\left(\overset{\left(i\right)}{\underset{1}{r}}\right).E_{d}\left(\overset{\left(i\right)}{\underset{2}{r}}\right).E_{c}(c^{\left(i\right)})\right)-s^{\left(i\right)}\right\|}_{2}$$ (2)

### 3.3 Incorporate dose-response curve

**Fig 3:**
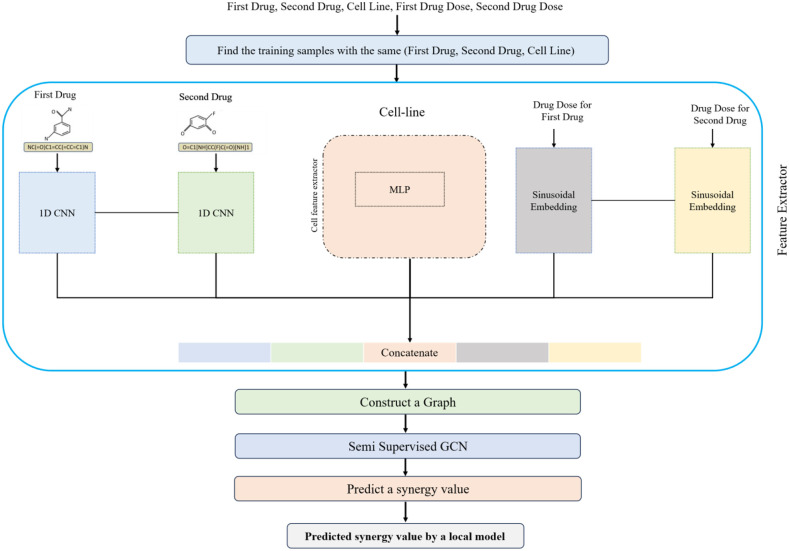
The overall schematic of the second module of the DeepDRP.

This step explains how the known values of the dose-response matrix for the input-paired drugs could be utilized in the proposed model. It should be noted that in the previous step, all the available data are used, while in this step, only known values of the dose-response matrix for the input paired drugs are used (if available). In other words, this step utilizes the input-related knowledge to predict the synergy value. The overall schematic of this step is shown in Fig 3.

In this step, a semi-supervised graph convolutional network is used. In the following, a brief explanation of the semi-supervised GCN is given, and then we will discuss how we utilized it in the proposed method.

#### 3.3.1 Semi-supervised GCN.

A Graph Convolutional Network (GCN) [39,40] is a type of neural network designed to work with graph-structured data. Semi-supervised GCNs have been shown to be effective in various applications, such as node classification, graph classification, and graph generation. It uses convolutional layers to aggregate information from neighboring nodes in the graph, similar to how traditional convolutional neural networks (CNNs) aggregate information from neighboring pixels in an image. Semi-supervised learning is a type of machine learning where the model is trained using labeled and unlabeled data. The goal is to leverage all available knowledge to improve the model’s performance. This allows the model to learn from labeled and unlabeled data, improving its performance on the nodes.

A Semi-Supervised GCN combines the strengths of GCNs and semi-supervised learning. It is trained on a graph with several labeled and unlabeled nodes. The model learns to aggregate information from neighboring nodes using graph convolutional layers. The label propagation layer propagates labels from labeled nodes to unlabeled nodes. Finally, the classification layer predicts labels for the nodes.

In the second module of the proposed method, we search over the training set to retrieve all samples that share the same drug pairs and cell line as the input sample but with different doses. Assume k shows the number of retrieved samples. It should be noted that there are two scenarios: 1) there are more than one retrieved sample, and 2) there are lower than one retrieved sample. In the first scenario, a semi-supervised graph is constructed and fed into the semi-supervised GCN to train the model. Finally, the label of the unlabeled sample (i.e., input sample) is predicted. The second scenario has no predicted outputs using the semi-supervised GCN. In the following, the detailed steps of both scenarios are given.

In the first scenario, there are k retrieved training samples with the known synergy value and an input sample with the unknown response. These k+1 samples are fed into the graph construction step (see Fig 3). We need a node feature matrix and an adjacency matrix to construct a graph. The feature matrix is a matrix with k+1 rows, and each row represents the feature vector of the corresponding node. The adjacency matrix is a k+1×k+1 matrix in which (i,j)^th^ element shows that there is an edge between node i and node j or not. In the proposed approach, to describe each node, we fed the corresponding drug pairs and its doses along the cell line to the corresponding embedding networks in the first step; then, they are concatenated. To construct the adjacency matrix, the four nearest neighbors based on the drug doses are selected for each node, and the corresponding edge is set as connected.

In the second scenario, if the mono-drug dose-response curves are available, we multiply the curves to construct the drug combination’s response curve, and then, based on this information, the retrieval step is done. If the mono-drug dose-response curves are not available, we retrieve the samples using the input feature matrix (same as the input of the synergy prediction network).

### 3.4 Aggregation step

**Fig 4:**
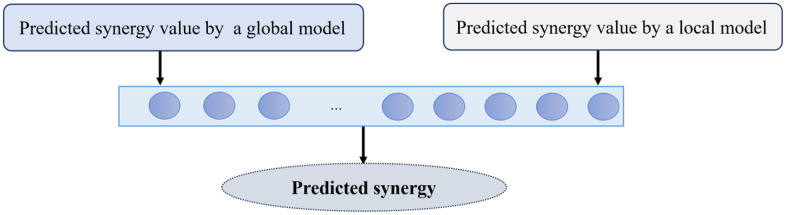
The overall schematic of the aggregation step of the DeepDRP.

This step aggregates the two different modules to predict the response value. It should be noted that the first module utilizes all available data to train the model. In other words, it utilizes the global knowledge (i.e., the global model), while the second module uses the corresponding known values of the dose-response curves for the input paired drugs (i.e., the local model). This step combines these two modules to predict the target variable. To do so, they are concatenated and fed into the multi-layer perceptron to predict the synergy value. The overall schematic of this module in the proposed method is given in Fig 4.

## 4 Experiments

The proposed method is evaluated in this section, and the obtained results are analyzed and discussed. To this end, the NCI-ALMANAC dataset and O’Neil dataset are used [41]. A detailed explanation of this dataset is given in the following. In this paper, to evaluate the proposed method, like similar approaches, three scenarios are designed: 1) in the first scenario (S1), the goal is to complete the incomplete dose-response matrices where only partial data is available, 2) the second scenario (S2) predicts the full dose-response matrix for new drug combinations with the available monotherapy response data for both individual drugs, and 3) the third scenario (S3) predicts the complete dose-response matrix for entirely new drug combinations that have no existing combination or monotherapy response data in any cell line.

The Pearson Correlation Coefficient (PCC) is used as an evaluation measure. It is a statistical measure that evaluates the strength and direction of the linear relationship between two continuous variables. In the context of predicting dose-response matrices, a high PCC value indicates that the predicted values are closely correlated with the actual values, suggesting a good model performance. PCC ranges from −1 (perfect negative correlation) to 1 (perfect positive correlation), with 0 indicating no correlation.

### 4.1 NCI-ALMANAC dataset

This paper uses the NCI-ALMANAC dataset to evaluate the proposed method, which encompasses drug combination dose responses in human cancer cells [41]. To enrich the data and ensure fair evaluation, our method, consistent with previously presented approaches, filtered the data to include cell lines with gene expression, CNV, CRISPR-Cas9 gene deletion, and proteomics data. This resulted in a comprehensive dataset containing drug combination and monotherapy response measurements in cancer cell lines originating from diverse tissue types. Each drug combination was meticulously screened using a dose-response matrix design, and response measurements were presented as percent growth, adhering to the NCI-60 standard test protocol. Notably, the combined drug response data distribution across cell lines mirrored the distribution observed in the broader NCI ALMANAC study.

Multi-omics data were sourced from the DepMap data portal to characterize the cell lines, encompassing gene expression, CNV, CRISPR-Cas9 gene deletion, and proteomics features. Given the substantial number of multi-omics features, computational feasibility dictated the selection of only the highest variance features across all cell lines, including gene expression, CNV, CRISPR-Cas9 gene deletion, and proteomics-related attributes. In our method, drug representation was standardized in SMILES format extracted using the PubChem API, ensuring consistency and compatibility with downstream analyses.

To have a fair comparison, like the comparable approaches, 5-fold cross-validation (CV) has been used to assess the model’s performance in three different scenarios. In 5-fold cross-validation, the data is randomly partitioned into five equal subsets. In each fold, four subsets (80% of the data) are used for model training, and the remaining one subset (20%) is held out for testing. This process is repeated five times, with each subset serving as the test set exactly once. The reported results are averaged across all five folds. This process has been repeated five times, with each part serving as the test set once. Three scenarios have been considered:

- Scenario S1: A subset of combination responses from each dose-response matrix has been randomly selected and used as the test set. The remaining data, including all monotherapy responses, has been used as the training set.
- Scenario S2: A subset of dose-response matrices for specific drug combinations in all cell lines has been randomly selected and used as the test set. All monotherapy responses have been kept in the training set.
- Scenario S3: This scenario has been set up similarly to S2, but all monotherapy responses have been excluded from the training set.

The hyperparameters are set through a grid search over a range of possible values. Specifically, the learning rate of the global model is searched over the range of 0.00001 to 0.1, while the learning rate of the local model is searched over the range of 0.000001 to 0.01. Additionally, the number of retrieved samples in the third scenario is set to {20, 50, 100}. These hyperparameters are chosen based on common values used in similar machine learning tasks and were tuned to achieve optimal performance.

#### 4.1.1 Results.

**Fig 5:**
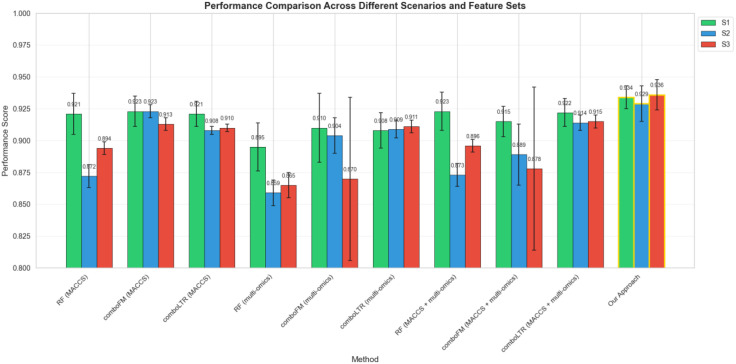
The obtained results of the proposed method and its comparison with the state-of-the-art approaches on NCI-ALMANAC dataset.

The results obtained are given in Fig 5, and they are compared with state-of-the-art approaches. Our methods improve the results compared to the other approaches. In scenario S1, our method gets a 1.2% improvement with respect to comboLTR. Also, our method achieves the most improvement in Scenario S3. We have achieved a 2.1% improvement over comboLTR. It should be noted that comboLTR uses many sources of auxiliary knowledge, including auxiliary chemical representation with MACCS fingerprint and multi-omics features, including gene expression, copy number variation, CRISPR-Cas9 gene knock-outs, and proteomics data. We have only utilized cell-line gene expression representation in the proposed approach as auxiliary knowledge.

The proposed aggregation mechanism is designed to adaptively combine predictions from the global model and the local semi-supervised GCN. When sufficient dose-response measurements are available for a given drug combination and cell line (Scenarios S1 and S2), the local component captures fine-grained dose-response structure and complements the global prediction. In contrast, in Scenario S3, where no local dose-response observations are available, the aggregation mechanism naturally reduces to the global model. This behavior allows DeepDRP to leverage local information when available while maintaining robust performance in data-scarce scenarios.

Table 1 reports the exact p-values from paired t-tests comparing our proposed method against nine state-of-the-art approaches across three experimental scenarios (S1, S2, and S3). Each p-value represents the probability of observing the measured performance difference (or more extreme) under the null hypothesis that our method and the baseline perform equally well. Our approach significantly outperformed all state-of-the-art methods across all three scenarios, with the exception of two comparisons where improvements were not statistically significant (RF with MACCS+multi-omics in S1: p = 0.0684; comboFM with MACCS in S2: p = 0.2132). In S3, our method demonstrated significant improvements over all competing approaches (p < 0.05).

**Table 1:** Statistical significance analysis (p-values) comparing our approach against state-of-the-art methods across three experimental scenarios.

Method	S1	S2	S3
RF (MACCS)	0.0334	<0.0001	<0.0001
comboFM (MACCS)	0.0421	0.2132	0.0002
comboLTR (MACCS)	0.0114	0.0007	<0.0001
RF (multi-omics)	0.0002	<0.0001	<0.0001
comboFM (multi-omics)	0.0247	0.0021	0.0113
comboLTR (multi-omics)	0.0008	0.0012	0.0001
RF (MACCS + multi-omics)	0.0684	<0.0001	<0.0001
comboFM (MACCS + multi-omics)	0.0030	0.0012	0.0211
comboLTR (MACCS + multi-omics)	0.0256	0.0078	0.0004

### 4.2 O’Neil dataset

To further assess the generalizability of DeepDRP, we conducted additional experiments on the O’Neil dataset [42], an independent drug combination screening resource that has been widely used as an external validation benchmark in previous studies. This dataset originates from a different screening platform with distinct experimental protocols compared to NCI-ALMANAC.

The O’Neil dataset contains 92,208 drug combination experiments across 39 cancer cell lines and 38 drugs, with full 5 × 5 dose-response matrices [42]. Synergy scores are calculated using the Bliss independence model, which differs from the percent growth measurements provided in NCI-ALMANAC. It should be noted that no additional multi-omics features (CNV, CRISPR-Cas9, proteomics) were incorporated, maintaining consistency with our primary experimental setup.

#### 4.2.1 Results.

We evaluated DeepDRP under three scenarios that have become standard benchmarks for this dataset [43]:

- New combination: Prediction involves a triplet in which the cell line and both drugs have each appeared individually in the training data, but never together as a complete triplet.
- New drug: The triplet includes either a novel drug pair or a previously unseen drug–cell line association—specifically, at least one of the drugs has not been observed in combination with the given cell line during training.
- New cell line: At the time of prediction, the queried triplet contains a cell line that was absent from the training set. However, the drug pairs themselves may have been seen previously in other contexts.

Table 2 presents the performance comparison of DeepDRP against four state-of-the-art methods on the O’Neil dataset include comboLTR [36], combokR [44], combokR 2.0 [43]. Results for baseline methods are reproduced under identical experimental conditions.

**Table 2:** Performance comparison (Pearson correlation coefficient) on O’Neil dataset across three transfer learning scenarios.

Method	New combo	New drug	New cell-line
comboLTR_mv	0.908	0.869	0.838
comboLTR_1v	0.902	0.867	0.825
combokR	0.937	0.907	--
combokR 2.0	0.939	0.935	0.938
Our approach	0.941	0.935	0.942

DeepDRP achieves state-of-the-art performance across all three evaluation scenarios, with Pearson correlation coefficients of 0.941, 0.935, and 0.942 for new combination, new drug, and new cell-line prediction, respectively. DeepDRP demonstrates the largest improvement in the most challenging scenario—predicting drug combination synergy in entirely unseen cell lines. Our approach achieves 0.942, substantially outperforming comboLTR_mv (0.838) and comboLTR_1v (0.825), and showing a + 0.004 gain over combokR 2.0 (0.938). This indicates that DeepDRP’s combination of global modeling and local dose-response graph learning enables better generalization to unobserved biological contexts.

### 4.3 Ablation study

To more thoroughly analyze the contribution of individual components in the proposed DeepDRP framework, we extend the ablation study beyond the coarse-grained global-only and local-only variants and evaluate the effect of key architectural modules independently. The goal of this analysis is to quantify how each design choice contributes to the overall performance across different data availability scenarios. Specifically, we consider the following model variants:

- Global-only (V1): This variant removes the local dose-response modeling module and relies solely on the global model trained on all available drug–dose–cell line combinations.
- Local-only (V2): In this setting, the global model is removed and predictions are made only using the local dose-response information derived from retrieved samples.
- w/o Dose Embedding: In this variant, the sinusoidal dose embedding module is replaced with raw normalized dose values, while keeping all other components unchanged.
- Local w/o GCN: This variant replaces the semi-supervised graph convolutional network in the local module with a simple multi-layer perceptron applied to the retrieved dose-response samples, thereby removing explicit graph-based neighborhood modeling.
- Fixed Aggregation: Instead of the learnable aggregation network, the predictions of the global and local modules are combined using a fixed averaging strategy.
- Full Model: The complete DeepDRP architecture, incorporating sinusoidal dose embedding, semi-supervised GCN-based local modeling, and learnable aggregation.

The obtained results are summarized in Table 3. As expected, the full DeepDRP model consistently achieves the best performance across all three evaluation scenarios.

**Table 3:** Ablation study results. The results obtained from the proposed method and two other variants show its effectiveness.

Methods	S1	S2	S3
Global-only (V1)	0.895 ± 0.021	0.861 ± 0.011	0.839 ± 0.009
Local-only (V2)	0.562 ± 0.021	0.510 ± 0.027	0.123 ± 0.031
w/o Dose Embedding	0.921 ± 0.013	0.912 ± 0.016	0.905 ± 0.014
Local w/o GCN (MLP)	0.907 ± 0.017	0.894 ± 0.019	0.876 ± 0.018
Fixed Aggregation (Average)	0.918 ± 0.015	0.909 ± 0.017	0.901 ± 0.016
***Full model***	**0.934 ± 0.009**	**0.929 ± 0.014**	**0.936 ± 0.012**

Removing the sinusoidal dose embedding results in a noticeable performance degradation, indicating that expressive dose representations are important for capturing nonlinear dose–response relationships. Similarly, replacing the semi-supervised GCN with a non-graph local predictor leads to reduced accuracy, demonstrating the benefit of explicitly modeling the geometric structure of dose-response neighborhoods. Moreover, the fixed aggregation strategy underperforms the learnable aggregation module, highlighting the importance of adaptive fusion of global and local predictions.

Among the coarse-grained variants, the global-only model (V1) performs reasonably well in data-scarce settings but fails to capture fine-grained dose-response structure, while the local-only model (V2) shows substantially lower performance, particularly in Scenarios S2 and S3, due to limited training signals. Overall, these results confirm that each component of DeepDRP contributes meaningfully to its predictive performance, and that the full architecture is necessary to achieve robust and accurate dose-response prediction across diverse experimental scenarios.

## 5 Discussion

In this paper, dose-response prediction for drug combinations is investigated. In this case, the system has five inputs: the first drug molecule and its corresponding dosage, the second drug and its dosage, and the cell line. The output is the synergy of these combinations. The proposed method predicts the synergy value using two sources of knowledge: global knowledge, which is achieved using all available training data, and local knowledge, which utilizes some of the training data with the same drug combination as the input. Then, these two levels of knowledge are aggregated to predict the final output.

In contrast to existing methods such as comboFM and comboLTR, which rely on a single global modeling strategy, DeepDRP uniquely leverages the local geometry of dose-response curves through a semi-supervised graph formulation, enabling improved generalization when experimental measurements are sparse.

One of the advantages of the proposed method is that it does not utilize hand-engineered feature extraction algorithms to represent the drug. However, it utilizes the learnable module (i.e., 1D convolutional neural network) to represent the drugs. Another advantage of the proposed method is that the semi-supervised GCN is utilized to consider the dose-response curve knowledge. It considers the neighboring doses to the input dosage and uses them to predict the synergy value for the input. One advantage of this model over classic regression models is that it does not require a specific functional form for the relationship between the predictor and response variables. In other words, our model does not assume a specific shape or form for the relationship, such as linear or quadratic, and instead allows the data to dictate the shape of the relationship. This contrasts with classic regression models, which typically assume a specific functional form for the relationship, such as a linear or logistic relationship. This can be a limitation of classic regression models, as the true relationship between the variables may not always follow the assumed form.

The ablation study further supports this analysis, showing that removing the local graph-based module or the adaptive aggregation mechanism leads to a noticeable performance drop, particularly in data-scarce scenarios, confirming that these components are responsible for the observed improvements.

While DeepDRP achieves improved prediction performance, it introduces additional computational steps compared to purely global models. Specifically, for each queried drug combination and cell line, a local dose-response graph is constructed using available experimental measurements. However, in practice these graphs are small, as they contain only the observed dose-response points for a single drug pair, typically resulting in tens of nodes. Consequently, both graph construction and semi-supervised GCN training incur limited computational overhead relative to global model inference.

Nevertheless, as the number of queried drug combinations grows, the cumulative cost of constructing and processing many local graphs may increase. Although these operations are embarrassingly parallel, further optimization or caching strategies may be required for deployment in large-scale or real-time settings. Addressing these scalability aspects will be an important direction for future work.
